# Health-related quality of life in Japanese patients with bladder cancer measured by a newly developed Japanese version of the Bladder Cancer Index

**DOI:** 10.1007/s10147-020-01770-2

**Published:** 2020-08-24

**Authors:** Takahiro Osawa, John T. Wei, Takashige Abe, Michitaka Honda, Shuhei Yamada, Jun Furumido, Hiroshi Kikuchi, Ryuji Matsumoto, Kazushi Hirakawa, Yasuyuki Sato, Yoshihiro Sasaki, Toru Harabayashi, Norikata Takada, Keita Minami, Hiroshi Tanaka, Ken Morita, Akira Kashiwagi, Naoto Miyajima, Tomoshige Akino, Sachiyo Murai, Yoichi M. Ito, Shunichi Fukuhara, Katsuhiko Ogasawara, Nobuo Shinohara

**Affiliations:** 1 Department of Urology, Hokkaido University Graduate School of Medicine, Sapporo, Japan; 2 Department of Urology, University of Michigan Health System, Ann Arbor, MI USA; 3 Department Minimally Invasive Surgical and Medical Oncology, Fukushima Medical University, Fukushima, Japan; 4 Department of Urology, Keiyukai Hospital, Sapporo, Japan; 5 Department of Urology, Kushiro Rosai Hospital, Kushiro, Japan; 6 Department of Urology, Hokkaido Cancer Center, Sapporo, Japan; 7 Department of Urology, Sapporo City General Hospital, Sapporo, Japan; 8 Department of Urology, Kushiro City General Hospital, Kushiro, Japan; 9 Department of Urology, Teine Keijinkai Hospital, Sapporo, Japan; 10 Clinical Research and Medical Innovation Center, Hokkaido University Hospital, Sapporo, Japan; 11 Section of Clinical Epidemiology, Department of Community Medicine, Kyoto University, Kyoto, Japan; 12 Department of General Medicine, Shirakawa STAR, Fukushima Medical University, Fukushima, Japan; 13 Graduate School of Health Sciences, Hokkaido University, Sapporo, Japan

**Keywords:** Bladder cancer, Quality of life, Bladder cancer index, Validation, Cancer survivor

## Abstract

**Introduction:**

We validated a Japanese version of the Bladder Cancer Index (BCI) as a tool for measuring health-related quality of life (HRQOL) in bladder cancer patients treated with various surgical procedures.

**Methods:**

The reliability and validity of the Japanese BCI were examined in 397 Japanese patients with bladder cancer via cross-sectional analysis. The patients simultaneously completed the Short Form (SF)-12, EQ-5D, and the Functional Assessment of Cancer Therapy-General and Bladder (FACT-G and FACT-BL). The differences in BCI subscales among various treatment groups were analyzed.

**Results:**

This study involved 397 patients (301 males and 96 females), with a mean age of 70 years and a median disease duration of 29 months (IQR: 12–66 months). Of these patients, 221 underwent transurethral resection of a bladder tumor, and 176 patients underwent radical cystectomy (ileal conduit: 101 patients, ileal neobladder: 49, and ureterostomy: 26). Cronbach’s alpha coefficient was ≥ 0.78 for all subscales, except the bowel bother subscale. Despite moderate correlations being detected between the function and bother score in urinary and bowel domains, the sexual function score was inversely correlated with the sexual bother score (r = − 0.19). A missing value percentage of > 15% was associated with old age (*p* < 0.05). The mean domain scores differed significantly among distinct clinically relevant treatment groups.

**Conclusions:**

Although revisions are needed to make it easier for elderly patients to comprehend, we confirmed the reliability and validity of the Japanese BCI. The Japanese BCI could be used for cross-cultural assessments of HRQOL in bladder cancer patients.

**Electronic supplementary material:**

The online version of this article (10.1007/s10147-020-01770-2) contains supplementary material, which is available to authorized users.

## Introduction

Performing HRQOL assessments in clinical practice has been demonstrated to not only increase health provider and patient awareness of treatment-related symptoms and enhance patient–physician communication, but also to improve patients’ overall survival [1]. Several studies have described the use of HRQOL assessments among Japanese patients that were treated for bladder cancer [2]. However, most of these HRQOL studies were performed using generic QOL instruments, such as the Medical Outcomes Short Form-36 (SF-36) and European Organization for Research and Treatment of Cancer Quality of Life Core Questionnaire (QLQ-C30) [3, 4]. Although these instruments can be used to assess HRQOL related to physical well-being and functional, emotional, and social factors after cancer treatment, they cannot necessarily be used to evaluate bladder cancer-specific side effects. Therefore, bladder cancer-specific HRQOL assessment instruments, such as the Functional Assessment of Cancer Therapy (FACT)-BL, have been used to evaluate HRQOL differences across various types of treatments [5, 6]. The main limitation of the FACT-BL is its inability to determine which symptoms are the most troublesome for a patient, because it does not break its questions into separate domains (e.g., into urinary, bowel, and sexual symptoms).

The Bladder Cancer Index (BCI) is bladder cancer-specific HRQOL instrument, which was designed as a comprehensive instrument for assessing HRQOL in bladder cancer patients who still possess their own bladders after surgery or who have undergone radical cystectomy [7]. The main advantage of the BCI is that it can be used to assess symptom magnitude and impairment in the urinary, bowel, and sexual health domains. Recently, the BCI was translated into Hungarian, Spanish, French, Dutch, and Arabian, and each of these versions has been validated [8–12]. To meet the demand for a bladder cancer-specific HRQOL questionnaire in Japan, we developed a preliminary Japanese version of the BCI through a multistep process, which involved forward-translation, back-translation, and discussion with the original developer. This Japanese BCI showed demonstrable face validity and reliability in the pilot study and can be used to estimate HRQOL among Japanese patients with bladder cancer and for cross-cultural comparisons [13]. This study was performed to validate the newly developed Japanese BCI using a large population of bladder cancer patients. Furthermore, we aimed to evaluate bladder cancer patients that had undergone various surgical procedures using the Japanese BCI via a multicenter cross-sectional survey conducted in Japan.

## Materials and methods

The validation cross-sectional cohort included bladder cancer patients with various disease stages (non-muscle-invasive and muscle-invasive cancer) that had undergone a range of surgical procedures (endoscopic treatment, intravesical therapy, or radical cystectomy). A total of 450 bladder cancer patients that were treated between 1999 and 2017 were enrolled from 7 institutions in Japan. Responses were obtained from 397 patients (88.2%). The study protocol was approved by each institutional review board, and the approval number for Hokkaido University Hospital was 015-0504. All patients were at least 20 years old and were required to have an Eastern Cooperative Oncology Group performance status (ECOG-PS) of 0 or 1. All patients provided written informed consent and responded to the relevant questionnaires, including the Japanese BCI, Medical Outcomes Short Form-12 (SF-12) [14], FACT-G [15], FACT-BL, and EQ-5D-5L [16]. All questionnaires were self-administered and sent back by mail or submitted to an outpatient clinic in a sealed envelope, so that privacy was maintained. The questionnaires were scored centrally by trained assessors.

### Item analysis, factor analysis, reliability, and validity procedures

The item analysis was performed using the data collected in the present study. The percentage of missing responses was calculated for each item. When ≥ 15% of the responses for an item were missing, the profiles of the responders and non-responders were compared using the Student’s t test or Pearson’s Chi square test. To identify discrete domains, we performed factor analysis using the principal component method with promax rotation. Each Likert scale item was categorized into one of 3 domains (urinary, bowel, or sexual) and 2 subdomains (urinary function/bother, bowel function/bother, and sexual function/bother). Each domain score was comprised of the mean of the standardized items. Higher scores indicate a better health status. The score for a domain was regarded as missing if more than 20% of the items in the domain were not answered. Other than that, the domain score was calculated from the answered questions. Cronbach’s alpha coefficient was used as a reliability coefficient for each subscale. To assess criterion-related validity, we analyzed the correlations between the scores for the BCI subscales and the scores for other relevant validated instruments (SF-12, FACT-G, FACT-BL, and EQ-5D-5L). Furthermore, to evaluate known-groups validity, the subjects were divided into the following 5 groups: (1) patients with native bladders that underwent transurethral resection alone, (2) patients with native bladders that underwent transurethral resection and intravesical therapy, (3) patients that underwent cystectomy involving ileal conduit diversion (IC), (4) patients that underwent cystectomy involving ileal neobladder diversion (NB), and (5) patients that underwent cystectomy involving cutaneous ureterostomy (CU). Statistical comparisons among the 5 groups were performed using the Kruskal–Wallis rank-sum test and the general linear model, followed by Tukey’s HSD test. Regarding intergroup comparisons, least-squares means (LSM) estimates, adjusted for age, sex, and ECOG-PS, were calculated. JMP version 14 (SAS Institute Inc., Cary, NC, USA) was used for the statistical analyses, and *p* values of < 0.05 were considered to indicate a statistically significant difference.

## Results

The median age, sex ratio, ECOG-PS, disease stage, and median disease duration of the validation cohort are shown in Table 1. Two hundred and twenty-one of the enrolled patients were treated endoscopically, including patients who underwent transurethral tumor resection with or without intravesical therapy. Of these patients, 104 had a history of one or more transurethral surgical procedures. Among those who received intravesical therapy, 73 patients and 30 patients were treated with intravesical BCG and intravesical chemotherapy (e.g., mitomycin C, epirubicin, pirarubicin), respectively. Of the other patients who underwent cystectomy, 101 (57.4% of those who were treated with cystectomy, 25.4% of the entire cohort) underwent IC, 49 (27.8% and 12.3%, respectively) underwent NB, and 26 (14.8% and 6.5%, respectively) underwent CU. The median disease duration for the native bladder without intravesical therapy group, native bladder with intravesical therapy group, cystectomy with IC group, cystectomy with NB group, and cystectomy with CU group were 22.5 months, 25 months, 35 months, 75 months, and 20.5 months, respectively. The percentage of missing items ranged from 2.3 to 33.0%. Eighteen of the 36 items exhibited a missing response rate of ≥ 15% (Supplementary Table 1). Among the 3 domains, the number of items with missing response rates of ≥ 15% was higher in the sexual domain (55.6%) than in the urinary domain (27.8%) or bowel domain (16.7%). Fifteen of the 18 missing items were presented in a table-style questionnaire, which matched the original version of the BCI; i.e., the missing items were presented in a table-style questionnaire format significantly more often than in an independent-style questionnaire format (*p *< 0.01). A higher number of missing responses for the 18 above-mentioned items was found to be associated with higher age, female sex, and a single marital status (Supplementary Table 1). The percentage of missing items ranged from 1.5% to 6.5% for SF-12, ranged from 2.0% to 2.5% for EQ-5D-5L, ranged from 5.0% to 48.6% for FACT-G, and ranged from 6.0% to 38.6% for FACT-BL, respectively.

**Table 1:** Clinical characteristics in 397 patients in Japanese version of the BCI validation cohort

	Endoscopy, no intravesical therapy	Endoscopy + intravesical therapy	Cystectomy + ileal conduit diversion	Cystectomy + orthotopic continent diversion	Cystectomy + ureterostomy diversion	Total no. (%)	p value
*n*	118	103	101	49	26	397	
Median age (IQR)	71 (64.75–77)	72 (66–79)	75 (65–78.5)	68 (64–73.5)	73.5 (66–81)		0.06
Sex:							0.11
Male	89 (75.4)	75 (72.8)	73 (72.3)	44 (89.8)	20 (76.9)	301 (75.8)	
Female	29 (24.6)	28 (27.2)	28 (27.7)	5 (10.2)	6 (23.1)	96 (24.1)	
ECOG performance status							<0.01
0	113 (95.8)	101 (98.1)	84 (83.2)	47 (95.9)	17 (65.4)	362 (91.2)	
1	2 (1.7)	1 (1.0)	13 (12.9)	2 (4.1)	9 (34.6)	27 (6.8)	
Unknown	3 (2.5)	1 (1.0)	4 (4.0)	0 (0.0)	0 (0.0)	8 (2.0)	
Living status							0.2
Married/partner	83 (70.3)	73 (70.9)	64 (63.4)	36 (73.5)	19 (73.1)	275 (69.3)	
Single	27 (22.9)	27 (26.2)	34 (33.7)	7 (14.3)	6 (23.1)	101 (25.4)	
Unknown	8 (6.8)	3 (2.9)	3 (3)	6 (12.2)	1 (3.8)	21 (5.3)	
Stage							<0.01
Ta, Tis, T1	111 (94.1)	101 (98.1)	51 (50.5)	21 (42.9)	10 (38.5)	294 (74.1)	
T2–T4	4 (3.4)	2 (1.9)	49 (48.5)	24 (49)	16 (61.5)	95 (23.9)	
Unknown	3 (2.5)	0 (0)	1 (1)	4 (8.2)	0 (0)	8 (2)	
CIS present	8 (6.8)	22 (21.4)	23 (22.8)	14 (28.6)	4 (15.4)	71 (17.9)	<0.01
Grade							<0.01
Low	90 (76.3)	44 (42.7)	13 (12.9)	5 (10.2)	3 (11.5)	155 (39)	
High	25 (21.2)	58 (56.3)	81 (80.2)	38 (77.6)	21 (80.8)	223 (56.2)	
Unknown	3 (2.5)	1 (1)	7 (6.9)	6 (12.2)	2 (7.7)	19 (4.8)	
Histology							0.07
UC	111 (94.1)	102 (99)	86 (85.1)	43 (87.8)	25 (96.2)	367 (92.4)	
Other	7 (5.9)	1 (1)	15 (14.9)	6 (12.2)	1 (3.8)	30 (7.6)	
Disease duration	22.5 (9.25–55.75)	25 (10–57)	35 (14.5–66.5)	75 (25.5–114)	20.5 (6–40.5)	29 (12–66)	<0.01

For all 3 domain scales and six domain subscales, Cronbach’s alpha coefficient was high (0.78–0.93), except for the bowel bother domain (0.64) (Table 2), indicating that each of the domain and subdomain items coherently measured urinary, bowel, or sexual factors. The percentage of patients reporting maximum scores was relatively high across all domains, indicating a moderate ceiling effect (Table 2). The ceiling effect was especially marked in the urinary and bowel domains, and the sexual bother domain. More than two-thirds of the patients who recorded the maximum score for each domain were endoscopically treated. Actually, the maximum score percentage was 100% for 5 out of 6 subdomains (Table 2). On the other hand, no floor effect was found, except for the urinary function (1.0%) and sexual function (35.0%) subdomains. Factor analysis supported the idea that most of the items could be assigned to urinary, bowel, or sexual health domains and their subdomains (their function and bother subscale), although there were two outliers (item numbers: 42 and 52) (Supplementary Table 2).

**Table 2:** BCI domain-specific summary and subscale characteristics

Domains	No. of Items	Scoring Minimum %	Scoring Maximum %	Floor effect %	Ceiling effect %	Chronbach’s α
Urinary						
Function	6	0	100	1.0%	56.2%	0.81
Bother	8	37.5	100	0.0%	31.7%	0.82
Bowel						
Function	4	12.5	100	0.0%	28.0%	0.78
Bother	6	33.3	100	0.0%	28.5%	0.64
Sexual						
Function	7	0	70.3	35.0%	0.0%	0.93
Bother	5	12.5	100	0.0%	48.6%	0.87

Floor effect: percentage of patients with worst possible score (0); ceiling effect: percentage of patients with best possible score (100)

The results of the assessments of the correlations between the domain scales and subscales are shown in Table 3. These correlations were evaluated to validate the convergence/divergence between function scores and bother scores. In each domain, the function scores and the bother scores exhibited moderate correlations (urinary and bowel subdomains: *r* = 0.42 and 0.47, respectively), indicating that bother subscale of each domain quantified similar disabilities to those measured by the respective function subscale. In the sexual domain, the function and the bother score exhibited a negative correlation, indicating that they were inversely correlated (*r* = − 0.19). In contrast, the correlations between the function subscale and the bother subscales of different domains were weak. Regarding concurrent validity, the SF-12 physical summary score and mental summary score were inversely correlated with the sexual bother and sexual function score, respectively (*r* = − 0.06 and − 0.11, respectively). In addition, the SF-12 role summary score and EQ-5D score were moderately correlated with the urinary bother score (*r* = 0.38 and *r* = 0.33, respectively). Despite moderate-to-strong correlations being detected between most of the BCI subdomain scores and the FACT-BL score (*r* = 0.32–0.48), the sexual bother subdomain score demonstrated a weak relationship with the FACT-BL score (*r* = 0.01). Table 4 shows that the urinary, bowel, and sexual domain scores differed significantly among the 5 treatment groups (*p* < 0.05), except for the sexual bother subdomain score (*p* = 0.23). However, regarding other HRQOL instruments, only the SF-12 role summary score (*p* < 0.01) and FACT-BL score (*p* < 0.01) differed significantly among the five groups.

**Table 3:** Interscale correlation between BCI function and bother subscales, and other HRQOL instrument summary scores

	Urinary		Bowel		Sexual	
Urinary	Function	Bother	Function	Bother	Function	Bother
Function	1.0					
Bother	**0.42**	1.0				
Bowel						
Function	0.21	0.33	1.0			
Bother	0.28	0.42	**0.47**	1.0		
Sexual						
Function	0.14	0.11	0.00	0.08	1.0	
Bother	0.18	0.26	0.10	0.25	**− 0.19**	1.0
SF-12 composite						
Physical summary	0.01	0.14	0.07	0.27	0.29	**− **0.06
Mental summary	0.01	0.24	0.16	0.21	**− **0.11	0.15
Role summary	0.16	0.38	0.14	0.28	0.15	0.04
Equation 5D_Score	0.15	0.33	0.22	0.41	0.16	0.09
FACT-G domain	0.06	0.28	0.15	0.23	0.07	0.02
FACT-BL	0.32	0.48	0.35	0.34	0.33	0.01

The underlined numbers mean the interscale correlation coefficients comparing function and bother scores within each domain (e.g., urinary function compared the urinary bother)

**Table 4:** Age, sex, and ECOG-PS adjusted Mean BCI domain scores by treatment group

	Mean native bladder without intravesical therapy	Mean native bladder with intravesical therapy	Mean cystectomy ileal conduit	Mean cystectomy neobladder	Mean cystectomy ureterostomy	*p* value
Urinary						
Function	94.52	95.26	89.68	53.12	96.18	<0.01
Bother	96.17	94.64	86.17	84.06	83.67	<0.01
Bowel						
Function	91.24	90.41	86.20	76.53	93.29	<0.01
Bother	92.90	92.43	87.51	84.41	91.55	<0.01
Sexual						
Function	17.20	17.82	5.46	7.06	3.49	<0.01
Bother	93.96	95.67	91.87	87.96	91.91	0.24
SF-12 composite						
Physical summary	46.51	47.21	48.43	47.87	39.10	0.02
Mental summary	54.05	55.15	54.87	54.68	56.98	0.70
Role summary	47.27	47.94	41.90	43.39	35.40	<0.01
Equation 5D_score	0.87	0.88	0.86	0.87	0.81	0.09
FACT-G domain	77.54	77.49	80.06	78.33	71.06	0.20
FACT-BL	33.23	32.65	31.10	29.96	30.52	<0.01

**Fig. 1:**
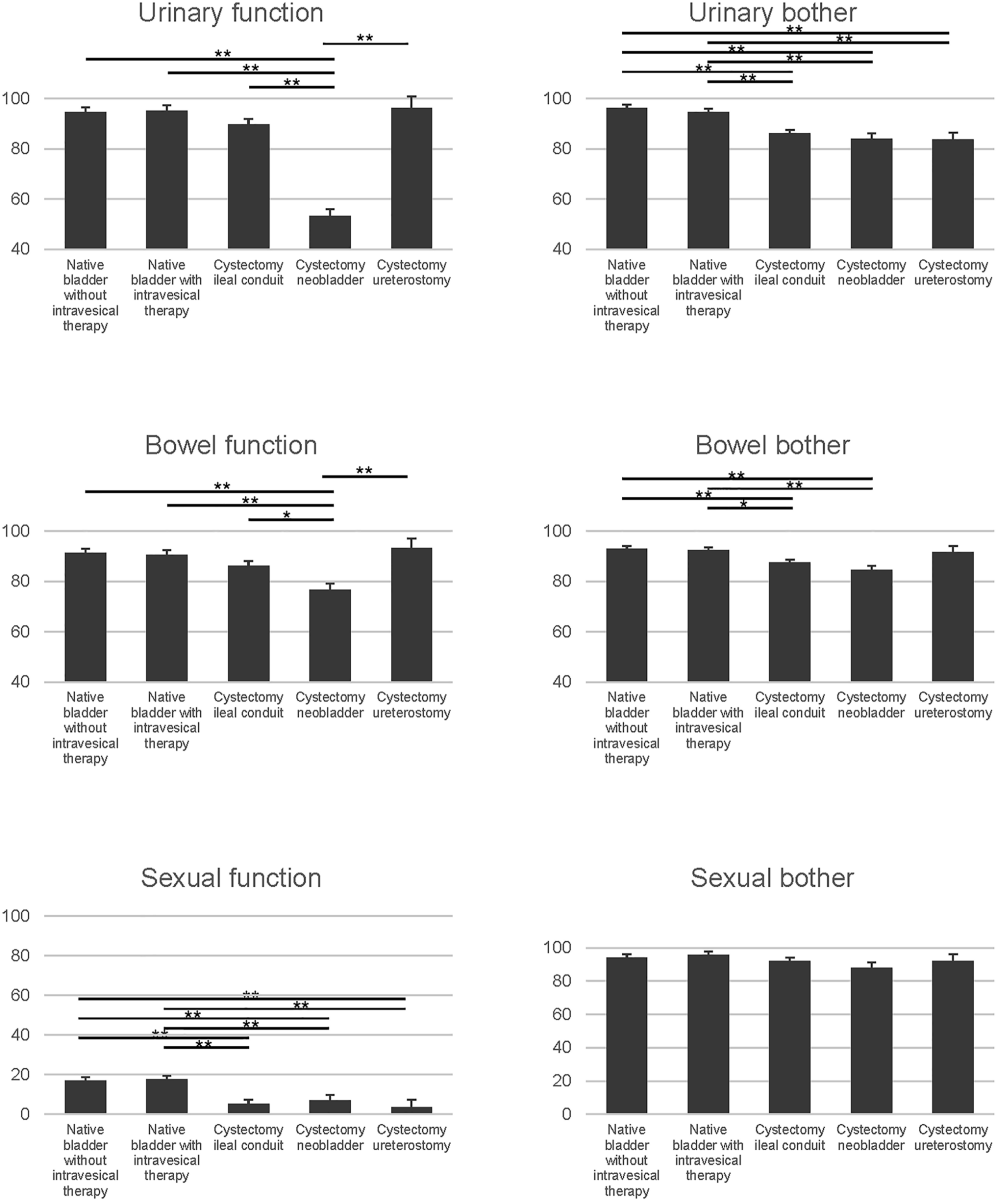
The least-squares means (LSM) estimation adjusted by age, sex, and ECOG-PS and standard error by treatment group (*n* = 371, Japanese cohort). Higher scores indicate better health status. (**indicates *p* < 0.05* and indicates *p* < 0.05.)

The mean BCI subscale scores for the 5 treatment groups, which were adjusted according to age, sex, and ECOG-PS, are shown in Fig. 1. Interestingly, the urinary and bowel function scores of the patients in the NB group were significantly lower than those of the patients in the other groups. In addition, the bowel bother score in patients that underwent cystectomy with IC or NB were significantly lower than those of the patients with native bladders (regardless of whether intravesical therapy was performed). Although the urinary bother scores and sexual function scores of the patients in the three cystectomy groups were significantly lower than those of the patients with native bladders, the sexual bother scores of the five treatment groups did not differ significantly. Regarding the FACT-BL score, it only differed significantly between the patients in the native bladder without intravesical therapy group and those in the radical cystectomy with IC group. The SF-12 physical summary scores of the patients in the CU group were significantly lower than those of the patients in the other cystectomy groups and the native bladder without intravesical therapy group (Supplementary Fig. 1). The SF-12 role summary scores of the patients in the IC group and CU groups were significantly lower than those of the patients with native bladders (Supplementary Fig. 1). Among the patients with native bladders, neither the number of prior transurethral surgical procedures nor the type of intravesical therapy significantly affected their BCI subdomain scores. In addition, the five treatment groups were divided into two groups by the median follow-up period, respectively: (i) the short-term follow-up group and (ii) the long-term follow-up group. The sexual bother score for the native bladder without intravesical group, the sexual function score for the NB group, and the bowel bother score for the IC group were significantly different between the short-term follow-up group and the long-term follow-up group (Supplementary Table 3). The QOL score for the long-term follow-up group was significantly higher compared with that of the short-term follow-up group in all the three domains.

## Discussion

In this study, we confirmed that the Japanese BCI can be used to comprehensively evaluate the postoperative symptom burdens of bladder cancer patients who undergo transurethral surgery or radical cystectomy. The Japanese BCI exhibited similar levels of reliability and validity to the original BCI. Substantial differences between patients that underwent different surgical treatments for bladder cancer were identified. In particular, we revealed HRQOL differences in the urinary, bowel, and sexual function and bother subdomains between patients that retained their native bladders and patients that underwent cystectomy using the Japanese BCI. To the best of our knowledge, this is the largest cross-sectional study to compare HRQOL among patients who have undergone surgical treatment for bladder cancer in Japan.

The current study highlights some important points that were not reported in the validation study of the original BCI. For 18 items of the BCI, a relatively high percentage of missing values (≥ 15%) was found to be associated with age, sex, and marital status. Therefore, BCI data obtained from elderly, female, and single patients should be interpreted carefully. Interestingly, the items that were presented in a table-style questionnaire format exhibited a high missing response rate. It is suggested that when completing table-style questionnaires elderly patients might find it difficult to see responses in the upper row when they answer questions in the lower rows. Consequently, we will revise the format of the table-style items in future. In addition, Japanese males have been reported to be reluctant to consult physicians about sexual issues, and therefore, might hesitate about answering items relating to sexual issues [17].

The relatively low internal consistency coefficient obtained for the bowel problem subdomain (Cronbach’s alpha coefficient: 0.64) in the present study is another issue. In a study involving an American population, the original BCI indicated that Cronbach’s alpha coefficient for the bowel problems subdomain was as high as 0.87; however, another international validation study reported lower Cronbach’s alpha coefficients for the bowel bother subdomain (range: 0.69–0.77) [9, 10]. One of the reasons for this is the weak correlations between the severity of constipation and other bowel bother items (e.g., pelvic pain, bloody stools, and bowel movement urgency). In addition, a high ceiling effect was detected in the patients who retained their native bladders. These findings were comparable to those obtained in the previous studies [10, 11], but suggested that the questionnaires should be modified, so that they can assess patients with good function more precisely. The conceptual independence among the urinary, bowel, and sexual domains was supported by the low inter-scale correlation coefficients for these relationships (range 0.05–0.32), which were very similar to those reported for the original BCI (range 0.17–0.39). The low correlations seen between the BCI and the SF-12 suggest that the BCI captures additional information that is not covered by generic instruments.

Mixed HRQOL results have been obtained regarding about whether continent diversion is superior or equivalent to incontinent diversion. Although several studies have reported that continent diversion provides marginal HRQOL advantages over incontinent diversion, most studies have shown no such differences [2, 18]. We found that patients who had undergone cystectomy with NB exhibited significantly lower HRQOL scores for the urinary function and bowel function subdomains than those who were treated with the other two incontinent diversion procedures. This suggests that urine leakage occurred more often and bowel movement frequency was increased in the patients that underwent NB than in those that underwent the other diversion procedures. However, the scores for urinary and bowel bother did not differ significantly among the cystectomy groups. In a longitudinal study, Hedgepeth also reported that patients that underwent NB exhibited significant functional urinary impairment with little difference in urinary problems [19].

Interestingly, the incontinent CU group and the incontinent IC group displayed comparable scores for most of the BCI subdomains. The previous studies also showed that the HRQOL scores of the patients that underwent CU did not differ significantly from those of the patients that underwent IC [4, 20]. Although CU is employed less frequently than other forms of urinary diversion due to the risk of stomal stenosis, it was reported that a high ureteral patency rate (82–89%) was achieved with CU using the Toyoda method [21, 22]. In addition, CU can be performed simply and safely and involves a shorter operating time and a lower bowel complications rate [20]. This procedure has generally been considered for elderly and frail patients with limited life expectancy. Possessing a single kidney is a good indication for CU, as we found that most of the patients (91%) who underwent cystectomy with simultaneous nephroureterectomy chose CU. CU is a reasonable alternative option for patients who are elderly or frail, based on the risk of perioperative complications and from the viewpoint of HRQOL.

The negative correlation between the sexual function and bother subscales deserves a mention (correlation coefficient: -0.19). One of the reasons for this negative correlation is due to about one-third of the patients who reported no sexual function without sexual bother. Although the Japanese patients in the current study displayed relatively low scores for the sexual function subdomain, they accepted their impairments rather than regarding them as bother. As was reported by Hisasue et al., satisfactory sexual function, such as erectile rigidity, does not always equate to a satisfactory sexual life in Japanese couples. These cultural factors might have influenced the discrepancy between the sexual function and sexual bother subdomains among Japanese patients. While a moderate correlation (correlation coefficient: 0.56) was detected between the sexual function and sexual bother subdomains in the original BCI study, weak relationships were detected between these subdomains in validation studies of the Spanish (correlation coefficient: 0.15) and Hungarian (correlation coefficient: 0.26) versions of the BCI [9, 11]. It is speculated that ethnicity and cultural differences might play significant roles in the perception of sexual impairment.

This study had several potential limitations. First, it lacked a baseline assessment, as it was a cross-sectional study, and data about how the patients’ HRQOL scores changed over time. However, this cross-sectional design also provides the potential advantage, because the patients with variety of follow-up periods can be analyzed. The analysis of such patients with the diversity of the follow-up periods enabled us to appropriately validate the Japanese BCI from various points of view. Second, our study did not involve any test–retest reliability testing; however, the observed high internal consistency of the BCI indicates that the results are reliable. The high proportion of missing values in the sexual domain was another limitation. In addition, other critical HRQOL factors, including patients’ body image perceptions, were not evaluated in this study. Finally, the cohorts were not random samples, and selection bias might have been unavoidable. The longer disease duration for the NB group compared with that of other four treatment groups should be considered when interpreting the HRQOL outcome of the NB group. Nevertheless, to the best of our knowledge, this is the first validation study of the Japanese BCI and the largest study to have compared HRQOL among patients that underwent surgical treatment for bladder cancer in Japan. We consider that the present Japanese BCI would contribute to evaluations of HRQOL in Japanese bladder cancer patients and provide considerable amounts of information that could be used to compare the outcomes of different surgical procedures.

The newly developed Japanese BCI was validated in bladder cancer patients that underwent surgery. Although it has several limitations, it will be a valuable tool for assessing the HRQOL of bladder cancer patients across a range of new treatment options, not only in clinical practice, but also during research. Further HRQOL-related research using the Japanese BCI, involving larger longitudinal bladder-cancer cohorts, would provide critical information and help clinicians to better understand the outcomes experienced by bladder cancer survivors.

## Electronic supplementary material

Below is the link to the electronic supplementary material.

Supplementary material 1 (DOCX 19 kb)

Supplementary material 2 (DOCX 19 kb)

Supplementary material 3 (DOCX 19 kb)
