# Supplementary material for: Health-related quality of life in Japanese patients with bladder cancer measured by a newly developed Japanese version of the Bladder Cancer Index
Source: Int J Clin Oncol. 2020 Aug 24;25(12):2090–8. doi: 10.1007/s10147-020-01770-2 (PMC7677272; doi:10.1007/s10147-020-01770-2)
Supplement: Supplementary file 1 — Supplementary material 1 (DOCX 19 kb) [file 10147_2020_1770_MOESM1_ESM.docx]

Supplementary table 1: Missing value 15% or greater vs age, sex, and living status

| BCI Item No. | Questions | No. Missing Values (%) | Age p value  (Student's t test) | Sex p value  (chi-square test) | Living status p-value  (chi-square test) |
| --- | --- | --- | --- | --- | --- |
| **URINARY DOMAIN** | |  |  |  |  |
| 7a)q28 | Urine leakage causing skin irritation | 17.1 | **<0.01** | 0.09 | 0.16 |
| 7b)q29 | Urine leakage causing body odor | 18.4 | **<0.01** | **0.02** | 0.36 |
| 7c)q30 | Blood in the urine | 19.1 | **<0.01** | 0.10 | 0.23 |
| 7d)q31 | Pain related to urination, stoma or catheterization | 19.9 | **<0.01** | 0.11 | 0.14 |
| 9b)q34 | Urinary trouble due to excercise | 15.4 | **<0.01** | **<0.01** | **0.045** |
| **BOWEL DOMAIN** | |  |  |  |  |
| 5b)q41 | Increased frequency of bowel movements | 16.1 | **<0.01** | **0.15** | 0.20 |
| 5c)q42 | Bloody stools | 18.6 | **<0.01** | 0.37 | 0.09 |
| 5d)q43 | Rectal/ Abdominal/ Pelvic pain | 17.4 | **<0.01** | 0.76 | 0.20 |
| **SEXUAL DOMAIN** | |  |  |  |  |
| 1b)q47 | Ability to reach orgasm | 20.7 | **<0.01** | **<0.01** | **<0.01** |
| 1c)q48 | Sensation in the genital area | 20.9 | **<0.01** | **<0.01** | **<0.01** |
| 1d)q49 | Ability to be sexually aroused | 19.6 | **<0.01** | **<0.01** | **<0.01** |
| 1e)q50 | Ability to have intercourse | 19.9 | **<0.01** | **<0.01** | **<0.01** |
| 3)q52 | Pain related to intercourse | 33.0 | **<0.01** | **<0.01** | **0.03** |
| **SEXAUL DOMAIN** | |  |  |  |  |
| 4a)q53 | Level of sexual desire | 17.9 | **<0.01** | **<0.01** | **<0.01** |
| 4b)q54 | Ability to have intercourse | 24.4 | **<0.01** | **<0.01** | **<0.01** |
| 4c)q55 | Ability to reach orgasm | 25.7 | **<0.01** | **<0.01** | **<0.01** |
| 5)q56 | Ability to function sexually | 17.6 | **<0.01** | **<0.01** | **<0.01** |
| 6)q57 | Lack of sexual function | 16.9 | **<0.01** | **<0.01** | **<0.01** |
