# Supplementary material for: Health-related quality of life in Japanese patients with bladder cancer measured by a newly developed Japanese version of the Bladder Cancer Index
Source: Int J Clin Oncol. 2020 Aug 24;25(12):2090–8. doi: 10.1007/s10147-020-01770-2 (PMC7677272; doi:10.1007/s10147-020-01770-2)
Supplement: Supplementary file 2 — Supplementary material 2 (DOCX 19 kb) [file 10147_2020_1770_MOESM2_ESM.docx]

Supplementary table 2:

| Item number | Domain | Item |
| --- | --- | --- |
| 24 | Urinary function domain | Over the past 4 weeks, how often have you leaked urine while awake and doing your normal activities? |
| 25 | Urinary function domain | Over the past 4 weeks, how often have you leaked urine while sleeping? |
| 26 | Urinary function domain | Over the past 4 weeks, which of the following best describes your urinary leakage when you are awake? |
| 27 | Urinary function domain | Over the past 4 weeks, which of the following best describes your urinary leakage when you are sleeping? |
|  |  | How big a problem, if any, has each of the following been for you during the past 4 weeks? |
| 28 | Urinary bother domain | a. Urine leakage causing skin irritation |
| 29 | Urinary bother domain | b. Urine leakage causing body odor |
| 30 | Urinary bother domain | c. Blood in the urine |
| 31 | Urinary bother domain | d. Pain related to urination, stoma or catheterization |
| 32 | Urinary bother domain | How big of a bother, if any, has your bladder, stoma, neo-bladder or catheterizable pouch been for you during the past 4 weeks? |
|  |  | Over the past 4 weeks, how much have difficulties with your bladder, stoma, neo-bladder or catheterizable pouch limited your activities? |
| 33 | Urinary bother domain | a. Social activities with friends |
| 34 | Urinary bother domain | b. Exercise |
| 35 | Urinary bother domain | c. Sleep |
| 36 | Bowel bother domain | How often have you had rectal urgency (felt like I had to pass stool, but did not) during the past 4 weeks? |
| 37 | Bowel function domain | How often have you had stools (bowel movements) that were loose or liquid (no form, watery, mushy) during the past 4 weeks? |
| 38 | Bowel bother domain | How often have your bowel movements been painful during the past 4 weeks? |
| 39 | Bowel function domain | How many bowel movements have you had on a typical day during the past 4 weeks? |
|  |  | How big a problem, if any, has each of the following been for you during the past 4 weeks? |
| 40 | Bowel function domain | a. Urgency to have a bowel movement |
| 41 | Bowel function domain | b. Increased frequency of bowel movements |
| 42 | Bowel bother domain | c. Bloody stools |
| 43 | Bowel bother domain | d. Rectal/ Abdominal/ Pelvic pain |
| 44 | Bowel bother domain | e. Constipation |
| 45 | Bowel bother domain | Overall, how big a problem has your bowel habits been for you during the past 4 weeks? |
|  |  | How would you rate each of the following during the past 4 weeks? |
| 46 | Sexual function domain | a. Your level of sexual desire? |
| 47 | Sexual function domain | b.Your ability to reach orgasm (climax)? |
| 48 | Sexual function domain | c. Your level of sensation in the genital area? |
| 49 | Sexual function domain | d. Your ability to be sexually aroused? |
| 50 | Sexual function domain | e. Your ability to have intercourse? |
| 51 | Sexual function domain | Over the past 4 weeks, how often did you have any sexual activity? |
| 52 | Sexual function domain | Over the past 4 weeks, how often have you had pain related to intercourse? |
|  |  | How big a problem, if any, has each of the following been for you during the past 4 weeks? |
| 53 | Sexual bother domain | a. Your level of sexual desire |
| 54 | Sexual bother domain | b. Your ability to have intercourse |
| 55 | Sexual bother domain | c. Your ability to reach orgasm |
| 56 | Sexual function domain | Overall, how would you rate your ability to function sexually during the past 4 weeks? |
| 57 | Sexual bother domain | Overall, how big a problem has your sexual function or lack of sexual function been for you during the past 4 weeks? |
