# Supplementary material for: Health-related quality of life in Japanese patients with bladder cancer measured by a newly developed Japanese version of the Bladder Cancer Index
Source: Int J Clin Oncol. 2020 Aug 24;25(12):2090–8. doi: 10.1007/s10147-020-01770-2 (PMC7677272; doi:10.1007/s10147-020-01770-2)
Supplement: Supplementary file 3 — Supplementary material 3 (DOCX 19 kb) [file 10147_2020_1770_MOESM3_ESM.docx]

Supplementary Table 3: The difference of the BCI subdomain score in the five groups between the short-term follow-up group (shorter than the median follow-up period) and the long-term follow-up group (longer than the median follow-up period). Wilcoxon test was performed for comparing the subdomain scores between the short-term follow-up group and the long-term follow-up

|  | Endoscopy, no intravesical therapy | | | Endoscopy + intravesical therapy | | | Cystectomy + ileal conduit diversion | | | Cystectomy + orthotopic continent diversion | | | Cystectomy + ureterostomy diversion | | |
| --- | --- | --- | --- | --- | --- | --- | --- | --- | --- | --- | --- | --- | --- | --- | --- |
| Median of disease duration (months) | 22.5 | | | 25 | | | 35 | | | 75 | | | 20.5 | | |
|  | Short-term follow-up (n=58) | Long-term follow-up (n=60) | p-value | Short-term follow-up (n=52) | Long-term follow-up (n=51) | p-value | Short-term follow-up (n=51) | Long-term follow-up (n=50) | p-value | Short-term follow-up (n=26) | Long-term follow-up (n=23) | p-value | Short-term follow-up (n=13) | Long-term follow-up (n=13) | p-value |
| **Urinary** |  |  |  |  |  |  |  |  |  |  |  |  |  |  |  |
| Function | 95.6 | 91.5 | 0.10 | 91.9 | 96.7 | 0.13 | 89.2 | 88.6 | 0.77 | 45.5 | 59.5 | 0.14 | 100.0 | 86.1 | 0.07 |
| Bother | 97.1 | 94.7 | 0.18 | 93.6 | 95.7 | 0.86 | 83.5 | 90.7 | 0.05 | 79.4 | 88.4 | 0.05 | 87.0 | 85.6 | 0.97 |
| **Bowel** |  |  |  |  |  |  |  |  |  |  |  |  |  |  |  |
| Function | 88.4 | 91.4 | 0.84 | 91.6 | 87.4 | 0.34 | 80.7 | 88.9 | 0.10 | 72.6 | 76.7 | 0.60 | 90.2 | 91.2 | 0.67 |
| Bother | 93.3 | 92.9 | 0.66 | 92.9 | 92.5 | 0.91 | 84.6 | 91.3 | **<0.01** | 83.0 | 88.0 | 0.23 | 93.0 | 90.7 | 0.65 |
| **Sexual** |  |  |  |  |  |  |  |  |  |  |  |  |  |  |  |
| Function | 21.6 | 17.8 | 0.23 | 21.2 | 17.2 | 0.44 | 6.8 | 8.3 | 0.72 | 6.2 | 18.2 | **0.02** | 5.2 | 2.1 | 0.77 |
| Bother | 89.4 | 95.4 | **0.01** | 94.4 | 94.4 | 0.76 | 90.9 | 91.5 | 0.63 | 84.3 | 87.2 | 0.90 | 88.0 | 97.5 | 0.33 |
